# Pioglitazone use associated with reduced risk of the first attack of ischemic stroke in patients with newly onset type 2 diabetes: a nationwide nested case–control study

**DOI:** 10.1186/s12933-021-01339-x

**Published:** 2021-07-27

**Authors:** Junghee Ha, Dong-Woo Choi, Keun You Kim, Chung Mo Nam, Eosu Kim

**Affiliations:** 1 Department of Psychiatry, Institute of Behavioral Science in Medicine, Yonsei University College of Medicine, 50-1 Yonsei-ro, Seodaemun-gu, Seoul, 03722 Republic of Korea; 2 Department of Biostatistics, Yonsei University Graduate School of Public Health, Seoul, South Korea; 3 Department of Preventive Medicine, Yonsei University College of Medicine, 50-1 Yonsei-ro, Seodaemun-gu, Seoul, 03722 Republic of Korea; 4 Brain Korea 21 FOUR Project for Medical Science, Yonsei University College of Medicine, Seoul, Republic of Korea

**Keywords:** Diabetes, Pioglitazone, Primary ischemic stroke, Population-based study

## Abstract

**Background:**

Pioglitazone use is known to be associated with a reduced risk of recurrent stroke in patients with diabetes mellitus (DM) who have a history of stroke. However, it is unclear whether this benefit extends to patients without a history of stroke. We aimed to evaluate the association between pioglitazone use and development of first attack of ischemic stroke in patients with newly diagnosed type 2 DM.

**Methods:**

Using longitudinal nationwide data from the 2002–2017 Korean National Health Insurance Service DM cohort, we analyzed the association between pioglitazone use and incidence of primary ischemic stroke using a nested case–control study. Among 128,171 patients with newly onset type 2 DM who were stroke-free at the time of DM diagnosis, 4796 cases of ischemic stroke were identified and matched to 23,980 controls based on age, sex, and the onset and duration of DM. The mean (standard deviation) follow-up time was 6.08 (3.34) years for the cases and controls. Odds ratios (ORs) and 95% confidence intervals (CIs) for the association between ischemic stroke and pioglitazone use were analyzed by multivariable conditional logistic regression analyses adjusted for comorbidities, cardiometabolic risk profile, and other oral antidiabetic medications.

**Results:**

Pioglitazone use was associated with a reduced risk of first attack of ischemic stroke (adjusted OR [AOR] 0.69, 95% CI 0.60–0.80) when compared with non-use. Notably, pioglitazone use was found to have a dose-dependent association with reduced rate of ischemic stroke emergence (first cumulative defined daily dose [cDDD] quartile AOR 0.99, 95% CI 0.74–1.32; second quartile, AOR 0.77, 95% CI 0.56–1.06; third quartile, AOR 0.51, 95% Cl 0.36–0.71; highest quartile, AOR 0.48, 95% CI 0.33–0.69). More pronounced risk reduction was found in patients who used pioglitazone for more than 2 years. A further stratified analysis revealed that pioglitazone use had greater protective effects in patients with risk factors for stroke, such as high blood pressure, obesity, and current smoking.

**Conclusions:**

Pioglitazone use may have a preventive effect on primary ischemic stroke in patients with type 2 DM, particularly in those at high risk of stroke.

**Supplementary Information:**

The online version contains supplementary material available at 10.1186/s12933-021-01339-x.

## Background

Type 2 diabetes mellitus (DM) is associated with increased mortality, which is primarily attributable to cerebrovascular disease [1, 2]. In particular, the risk of stroke is three times higher in patients with DM than that in the general population [3, 4]. Insulin resistance, a pivotal pathogenic condition found in DM, plays a critical role in the development of ischemic stroke via aggravation of atherosclerosis [5]. On the other hand, insulin resistance is also considered a modifiable risk factor in terms of stroke prevention [6].

Pioglitazone is an agonist of peroxisome proliferation activated receptor γ (PPAR-γ), and one of the most potent insulin-sensitizing drugs. Recent clinical trials have demonstrated its beneficial effects on cerebrovascular outcomes by mitigating the progression of carotid intima-media thickness independent of glycemic control [7, 8]. Previous clinical studies have provided strong evidence supporting the usefulness of pioglitazone for secondary stroke prevention [9, 10]. For instance, Kim et al. recently reported that treatment with pioglitazone significantly lowered the risk of recurrent stroke in patients with DM and acute ischemic stroke [10].

Despite extensive experimental studies demonstrating the potential benefit of pioglitazone use in cerebrovascular risk, large-scale clinical studies are limited to patients with a history of previous stroke, and the preventive effects of pioglitazone use against primary ischemic stroke are often conflicting. Although the Prospective Pioglitazone Clinical Trial in Macrovascular Events (PROactive) revealed that treatment with pioglitazone contributed to a decreased risk of stroke compared with placebo in patients with type 2 DM with previous stroke history, there was no significant preventive effect of pioglitazone use on the development of stroke in a subgroup without prior stroke [11]. Another study reported that pioglitazone use for less than 3 years increased the risk of ischemic stroke in elderly patients with DM, whereas a recent retrospective study found that pioglitazone use prevented new-onset ischemic stroke in patients with DM who had cardiovascular risk factors [12, 13]. However, these previous studies were conducted only with participants who had cardiovascular risk factors such as hypertension or hyperlipidemia; hence, their generalizability is limited. There has been no report of such beneficial effects of pioglitazone use against the first attack of ischemic stroke in the general population of patients with DM. In real-world practice, the effect of pioglitazone on stroke may vary according to different clinical characteristics in patients taking the medication and its interaction with other glucose-lowering agents.

Therefore, we aimed to examine the preventive effect of pioglitazone use on first attack of ischemic stroke in a general DM population using a nested case–control design. To account for confounding effects by indication, we assessed the cardiometabolic risk profile and prescription registry date of patients with newly diagnosed type 2 DM with no previous history of stroke.

## Methods

### Study design and data source

We used the 2002–2017 dataset from the Korean National Health Insurance Service (NHIS)-DM cohort. It contains data from 400,000 patients with type 2 DM, which corresponds to a sample of approximately 23% of the entire type 2 DM population in the 35–85-year age group in South Korea. This dataset includes all inpatient and outpatient medical claims data, including data on prescription drug use, diagnostic and treatment codes, and primary and secondary diagnosis codes. It also includes the National Health Screening Program (NHSP) data. Since 2000, the Korean government has implemented an obligatory NHIS, which covers up to 98% of the entire Korean population. All insured adults are eligible for the NHSP and are recommended to undergo a standardized health check-up every 1–2 years. The Korean NHIS claims database records diagnoses based on the International Statistical Classification of Disease and Related Health Problems, Tenth Revision (ICD-10) codes. This study was approved by the Institutional Review Board of Yonsei University Health System (approval no. 4-2021-0127), and the approving authority waived the requirement for informed consent because of the use of deidentified patient data.

### Selection of cases and controls

From the Korean NHIS-DM cohort, a total of 128,171 patients with newly diagnosed DM (ICD E11) with no previous history of ischemic stroke, who had undergone a health check-up between 2004 and 2012 were enrolled, and follow-up data collected until December 2017 were reviewed. Ischemic stroke was diagnosed using ICD-10 code I63. To ensure diagnostic accuracy, patients were defined as having ischemic stroke only when it was a primary diagnosis code on admission and when they underwent brain computed tomography, magnetic resonance imaging, or magnetic resonance angiography during hospitalization because of the assumption that those with acute ischemic stroke should undergo brain imaging [14]. We excluded (i) patients who had not used antidiabetic medications (n = 39,564); (ii) patients receiving insulin treatment for more than 30 days (n = 7,027); (iii) patients with the onset of any type of stroke (ICD I60–I64) within 30 days of DM diagnosis (n = 1234); (iv) cases with no matching control (n = 235); (v) patients exposed to rosiglitazone during the study period (n = 13,291). Patients treated with rosiglitazone were excluded to avoid the confounding effect of rosiglitazone, another drug of the thiazolidinedione class withdrawn from the Korean market in 2010 due to increased cardiovascular risk concerns. The index date was defined as the date of ischemic stroke diagnosis. Control subjects were randomly selected at a 1:5 ratio from the DM cohort at the time when the case subjects were selected and were matched based on age, sex, the time point of DM diagnosis, and DM duration using incident density sampling.

### Exposure to pioglitazone

Patients with total prescriptions for pioglitazone of ≥ 90 cumulative defined daily doses (cDDDs) after the onset of DM treatment were deemed pioglitazone users. This definition was applied for pioglitazone and other antidiabetic drugs including insulin, sulfonylurea, biguanide, dipeptidyl peptidase-4 inhibitor, and alpha-glucosidase as they may confound the data. To obtain the cDDD for each patient, we summed all the pioglitazone prescriptions dispensed during a defined period (i.e., from the onset of DM to the index date of the diagnosis of ischemic stroke) and then converted the quantity to the number of cDDDs according to the World Health Organization definition [15]. Pioglitazone exposure was described using three criteria: (i) ever-user; (ii) cDDD; and (iii) duration of the prescription. The cDDD and duration were classified by quartiles.

### Potential confounders

We obtained information on selected comorbid conditions from inpatient and outpatient hospital diagnoses. The incidence of hypertension, dyslipidemia, heart failure, arterial fibrillation, ischemic heart disease (IHD), and depression and the Adapted Diabetes Complications Severity Index (aDCSI) from DM diagnosis to the index date were estimated [16]. The Charlson Comorbidity Index (CCI) was measured during the 1 year before the DM diagnosis. Fasting blood glucose levels, systolic blood pressure, diastolic blood pressure, total cholesterol levels, creatinine levels, body mass index (BMI; < 18.5, 18.5–22.9, 23.0–25.0, and ≥ 25.0 kg/m^2^), smoking status (none, past, and current), alcohol consumption (low: < 1 time/week, moderate: 1–4 times/week, and heavy: 5–7 times/week), and physical activity (yes: ≥ 1 time/week; no: never) were measured as close as possible to the DM diagnosis date.

### Statistical analyses

The characteristics of the study population were analyzed descriptively using the standardized mean difference (SMD). SMD values above 0.2 were regarded as a potential imbalance between the two groups [17]. Conditional logistic regression analysis was conducted to investigate the association between pioglitazone use and ischemic stroke. We calculated the crude odds ratio (OR), adjusted OR (AOR), and 95% confidence interval (CI) for ischemic stroke between pioglitazone users and non-users. The analyses were adjusted for the following variables: hypertension, dyslipidemia, atrial fibrillation, heart failure, CCI, aDCSI, depression, fasting blood glucose levels, systolic blood pressure, diastolic blood pressure, total cholesterol levels, creatinine levels, statin use, cardiovascular medications (aspirin, statin, anticoagulant, antiplatelet, and antihypertension drugs), other antidiabetic medications, BMI, alcohol and smoking habits, and physical activity. To further assess the potential influence of preexisting risk factors for stroke, such as age, sex, hypertension, dyslipidemia, atrial fibrillation, obesity, smoking, alcohol consumption, and no physical activity, we stratified the analyses to assess people with risk factors and without risk factors separately. We used incident density random sampling for our primary analysis; however, the random imbalance of covariates between pioglitazone users and non-users may have affected the main outcome. Hence, we further conducted propensity score-matching (PSM) to control for covariate imbalance that could potentially lead to selection bias. We conducted PSM using the nearest neighbor matching method and baseline covariates in a 1:4 ratio. A *p*-value < 0.05 was considered significant. All statistical analyses were performed using SAS software, version 9.4 (Cary, NC, USA).

## Results

### Study population

**Fig. 1:**
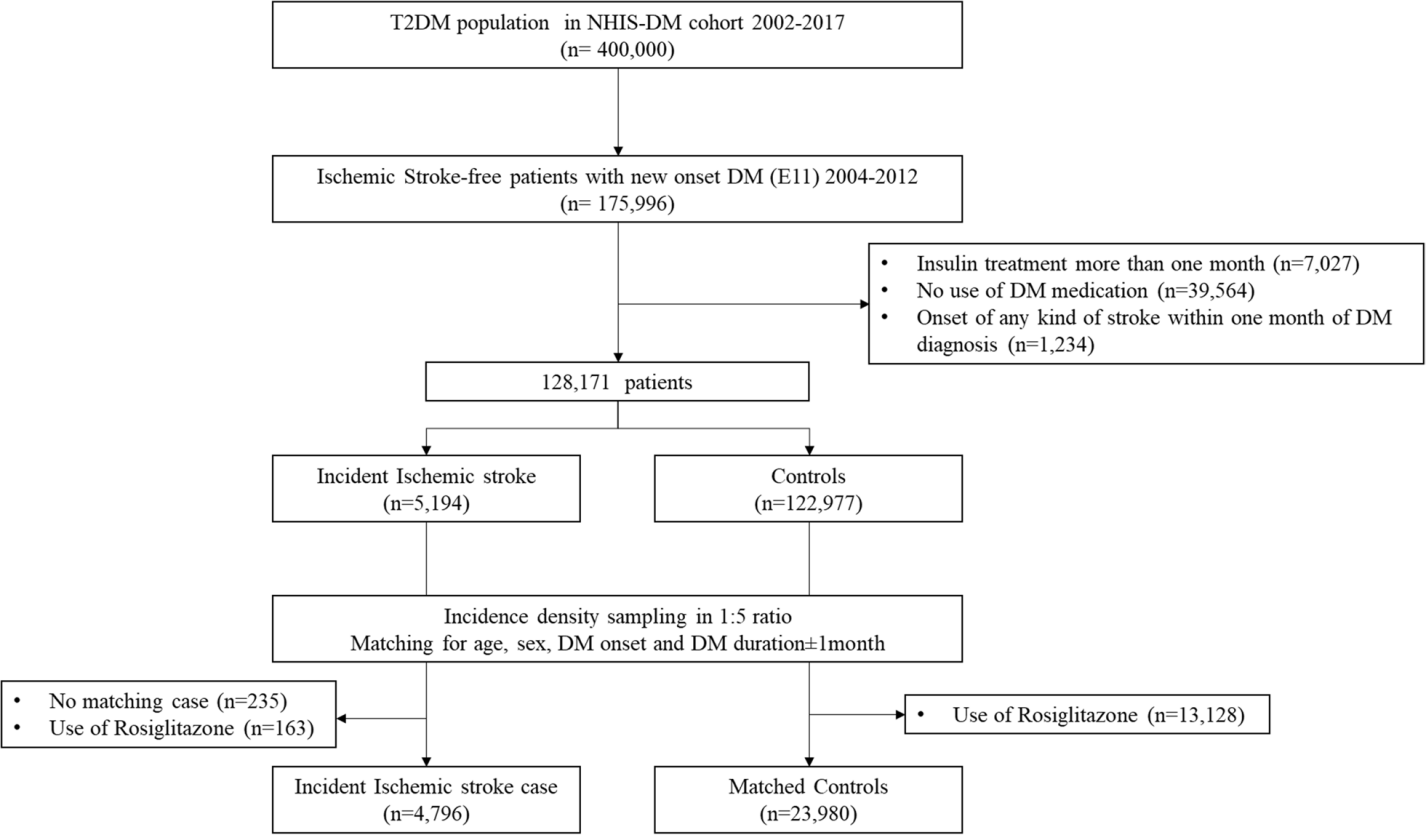
Flow chart of the study design

A total of 128,171 patients with newly diagnosed type 2 DM with no history of stroke were included in the study. Of these patients, we identified 4,796 ischemic stroke cases and matched them with 23,980 controls (Fig. 1). Table 1 shows the baseline characteristics of the cases and controls. Age, sex, date of DM diagnosis, and DM duration did not differ between the cases and controls after incidence density sampling. Compared with controls, cases were more likely to have hypertension, atrial fibrillation, heart failure, and depression. They were also more likely to use antihypertensive, antiplatelet, anticoagulant, and antiarrhythmic agents. The proportion of individuals with current smoking status and heavy alcohol intake was higher among cases than among controls. The individuals were less likely to be physically active.

**Table 1:** Baseline characteristics of patients with ischemic stroke (cases) and controls

Variables	Cases (n = 4796)	Controls (n = 23,980)	*SMD*
	n (%)	n (%)	
Age	61.88 ± 8.98	61.88 ± 8.98	< 0.001
Women	2034 (42.4)	10,170 (42.4)	< 0.001
Diabetes duration (years)	6.08 ± 3.34	6.08 ± 3.34	0.001
< 5 years	1929 (40.2)	9633 (40.2)	
5–10 years	2175 (45.4)	10,876 (45.4)	
≥ 10 years	692 (14.4)	3471 (14.5)	
BMI			0.035
< 18.5 kg/m^2^	59 (1.2)	210 (0.9)	
18.5–22.9 kg/m^2^	1002 (20.9)	5036 (2.10)	
23–25 kg/m^2^	1185 (24.7)	6008 (25.1)	
≥ 25 kg/m^2^	2550 (53.2)	12,726 (47.76)	
Fasting blood glucose (mg/dL)^a^	138.54 ± 53.91	134.89 ± 47.76	0.072
BP (mmHg)^a^			
Systolic	134.92 ± 17.91	132.38 ± 17.04	0.146
Diastolic	82.14 ± 11.37	80.83 ± 10.74	0.118
Total cholesterol (mg/dL)^a^	205.23 ± 42.81	203.90 ± 41.61	0.032
Creatinine (mg/dL)^†^	1.02 ± 0.86	1.03 ± 0.95	0.005
Hypertension	3922 (81.8)	17,998 (78.1)	0.164
Atrial fibrillation	339 (7.1)	728 (3.0)	0.185
Ischemic heart disease	1269 (26.5)	5571(23.2)	0.075
Heart failure	732 (15.3)	2552 (10.6)	0.138
Dyslipidemia	2,446 (51.0)	13,484 (56.2)	0.105
CCI			0.03
0	1442 (30.1)	7059 (29.4)	
1	1156 (24.1)	5583 (23.3)	
2	2198 (45.8)	11,338 (47.3)	
aDCSI			0.147
0	4175 (87.1)	21,951 (91.5)	
1	445 (9.3)	1402 (5.8)	
2	176 (3.7)	627 (2.6)	
Depression	663 (13.8)	2,969 (12.4)	0.043
Medication			
Statin	2589 (54.0)	13,445 (56.1)	0.042
Aspirin	2788 (58.1)	12,741 (53.1)	0.101
Antiplatelet	961 (20.0)	2991 (12.5)	0.206
Anticoagulant	204 (4.3)	438 (1.8)	0.142
Antihypertensive agents	3551 (74.0)	16,150 (67.3)	0.147
Antiarrhythmic agents	551 (11.5)	2188 (9.1)	0.078
Antidiabetic medication			
Biguanide	3813 (79.5)	17,598 (73.4)	0.145
Alpha-glucosidase inhibitors	411 (8.6)	1716 (7.2)	0.053
DPP- IV inhibitors	1220 (25.4)	4721 (19.7)	0.138
Insulin	1983 (41.3)	5177 (21.6)	0.435
SGLT2 inhibitors	47 (1.0)	174 (0.7)	0.028
Sulfonylurea	3550 (74.0)	15,972 (66.6)	0.163
Smoking			
None	2963 (61.8)	15,772 (65.8)	0.154
Past	570 (11.9)	3399 (14.2)	
Current	1263 (26.3)	4809 (20.1)	
Alcohol use			0.056
Low	3497 (72.9)	17,561 (73.2)	
Moderate	953 (19.9)	5002 (20.9)	
Heavy	346 (7.2)	1417 (5.9)	
Physical activity			0.121
Yes (≥ 1 time per week)	3182 (66.3)	17,244 (71.9)	

*aDCSI* adapted Diabetes Complication Severity Index, *BP* blood pressure, *CCI* Charlson Comorbidity Index, *DPP-IV* dipeptidyl peptidase IV, *SGLT2* sodium-glucose co-transporter 2, *SMD* Standardized mean difference

^a^Mean and standard deviation of the continuous independent variables in this study

### Pioglitazone use and ischemic stroke development

The associations between pioglitazone use and ischemic stroke were evaluated using conditional logistic models (Table 2). During the study period, 246 patients with ischemic stroke (5.1%) and 1513 controls (6.3%) had used pioglitazone. In the univariate analysis, pioglitazone use was associated with lower odds for ischemic stroke (OR [CI], 0.79 [0.67–0.92]), compared with non-use. Upon conditional logistic regression analyses adjusted for medical comorbidities and treatment with oral antidiabetic medication, we found that pioglitazone use was significantly associated with a lower risk of primary ischemic stroke (adjusted OR 0.69, 95% CI, 0.60–0.80). Table 2 shows that pioglitazone use for extended periods has dose–response relationships with lowering risk of first onset ischemic stroke (< 171.5 cDDDs, adjusted OR 0.99; 95% CI, 0.74–1.32; 171.5–324 cDDDs, adjusted OR 0.77, 95% CI, 0.56–1.06; 325–576 cDDDs, adjusted OR 0.51, 95% CI, 0.36–0.71; ≥ 577 cDDDs, adjusted OR 0.48, 95% CI, 0.33–0.69). The greatest risk reduction was shown in pioglitazone users with the highest cDDDs (≥ 577) with an AOR of 0.48 (95% CI, 0.33–0.69), compared with non-users. Further investigation into the effect of duration of pioglitazone use showed a significant reduction in stroke risk in patients who used pioglitazone for 2 years or more (Table 3).

**Table 2:** Reduced ischemic stroke risk associated with pioglitazone use in diabetes mellitus patients

	Cases (n = 4,796)	Controls (n = 23,980)	Crude OR (95% Cl)	*p*-value	AOR (95% Cl)*	*p*-value
	n (%)	n (%)				
Pioglitazone use						
Never user	4550 (94.9)	22,467 (93.7)	1 (reference)		1 (reference)	
Users	246 (5.1)	1513 (6.3)	0.79 (0.67–0.92)	0.002	0.69 (0.59–0.80)	< 0.0001
Cumulative dose of use						
Never user	4550 (94.9)	22,467 (93.7)	1 (reference)		1 (reference)	
Ever-user						
Q1 (< 171.5 cDDDs)	85 (1.8)	356 (1.5)	1.17 (0.89–1.53)	0.253	0.99 (0.74–1.32)	0.938
Q2 (171.5–324 cDDDs)	68 (1.4)	371 (1.5)	0.90 (0.67–1.20)	0.469	0.77 (0.56–1.06)	0.103
Q3 (325–576 cDDDs)	50 (1.0)	390 (1.6)	0.63 (0.46–0.87)	0.005	0.50 (0.36–0.70)	< 0.001
Q4 (≥ 577 cDDDs)	43 (0.9)	396 (1.7)	0.50 (0.36–0.71)	< 0.001	0.48 (0.33–0.69)	< 0.001

*AOR* adjusted odds ratio, *cDDDs* cumulative defined daily doses, *CI* confidence interval, *Q* quartile

^*^Analysis was adjusted for the following covariates: hypertension, ischemic heart disease, dyslipidemia, Charlson Comorbidity Index, Diabetes Complications Severity Index, depression, statin use, aspirin use, antiplatelet use, anticoagulant use, antihypertensive drug use, antiarrhythmic drug use, use of antidiabetic medications, fasting blood glucose levels, systolic blood pressure, diastolic blood pressure, total cholesterol levels, creatinine levels, body mass index, smoking status, alcohol consumption, and physical activity

**Table 3:** Relationship of pioglitazone use to primary ischemic stroke stratified by duration of pioglitazone use in patients with type 2 diabetes

	Cases (n = 4796)	Controls (n = 23,980)	Crude OR (95% Cl)	*p*-value	AOR (95% Cl)*	*p*-value
	n (%)	n (%)				
Duration of use (days, quartile)						
Never user	4,550 (94.9)	22,467 (93.7)	1 (reference)		1 (reference)	
Ever-user						
Q1 (< 336 days)	84 (1.8)	354 (1.5)	1.15 (0.88–1.51)	0.253	0.96 (0.72–1.29)	0.807
Q2 (227–630 days)	68 (1.4)	364 (1.5)	0.93 (0.70–1.25)	0.469	0.78 (0.57–1.07)	0.220
Q3 (631–1,129 days)	55 (1.2)	394 (1.6)	0.68 (0.50–0.92)	0.005	0.54 (0.39–0.75)	< 0.001
Q4 (≥ 1,130 days)	39 (0.8)	401 (1.7)	0.46 (0.32–0.65)	< 0.001	0.45 (0.31–0.65)	< 0.001
Duration of use (years)						
Never user	4550 (94.9)	22,467 (93.7)	1 (reference)		1 (reference)	
Ever-user						
Less than 1 year	91 (1.9)	410 (1.7)	1.08 (0.83–1.40)	0.253	0.91 (0.69–1.19)	0.480
1–2 years	75 (1.6)	425 (1.8)	0.86 (0.65–1.14)	0.469	0.72 (0.53–0.97)	0.028
2–3 years	37 (0.8)	260 (1.1)	0.72 (0.50–1.05)	0.005	0.56 (0.38–0.84)	0.004
More than 3 years	43 (0.9)	418 (1.7)	0.48 (0.34–0.67)	< 0.001	0.46 (0.32–0.67)	< 0.001

*AOR* adjusted odds ratio, *CI* confidence interval, *Q* quartile

^*^Analysis was adjusted for the following covariates: hypertension, ischemic heart disease, dyslipidemia, Charlson Comorbidity Index, Diabetes Complications Severity Index, depression, statin use, aspirin use, antiplatelet use, anticoagulant use, antihypertensive drug use, antiarrhythmic drug use, use of antidiabetic medications, fasting blood glucose levels, systolic blood pressure, diastolic blood pressure, total cholesterol levels, creatinine levels, body mass index, smoking status, alcohol consumption, and physical activity

### The protective effect of pioglitazone on first stroke was more evident in people at risk for stroke

**Fig. 2:**
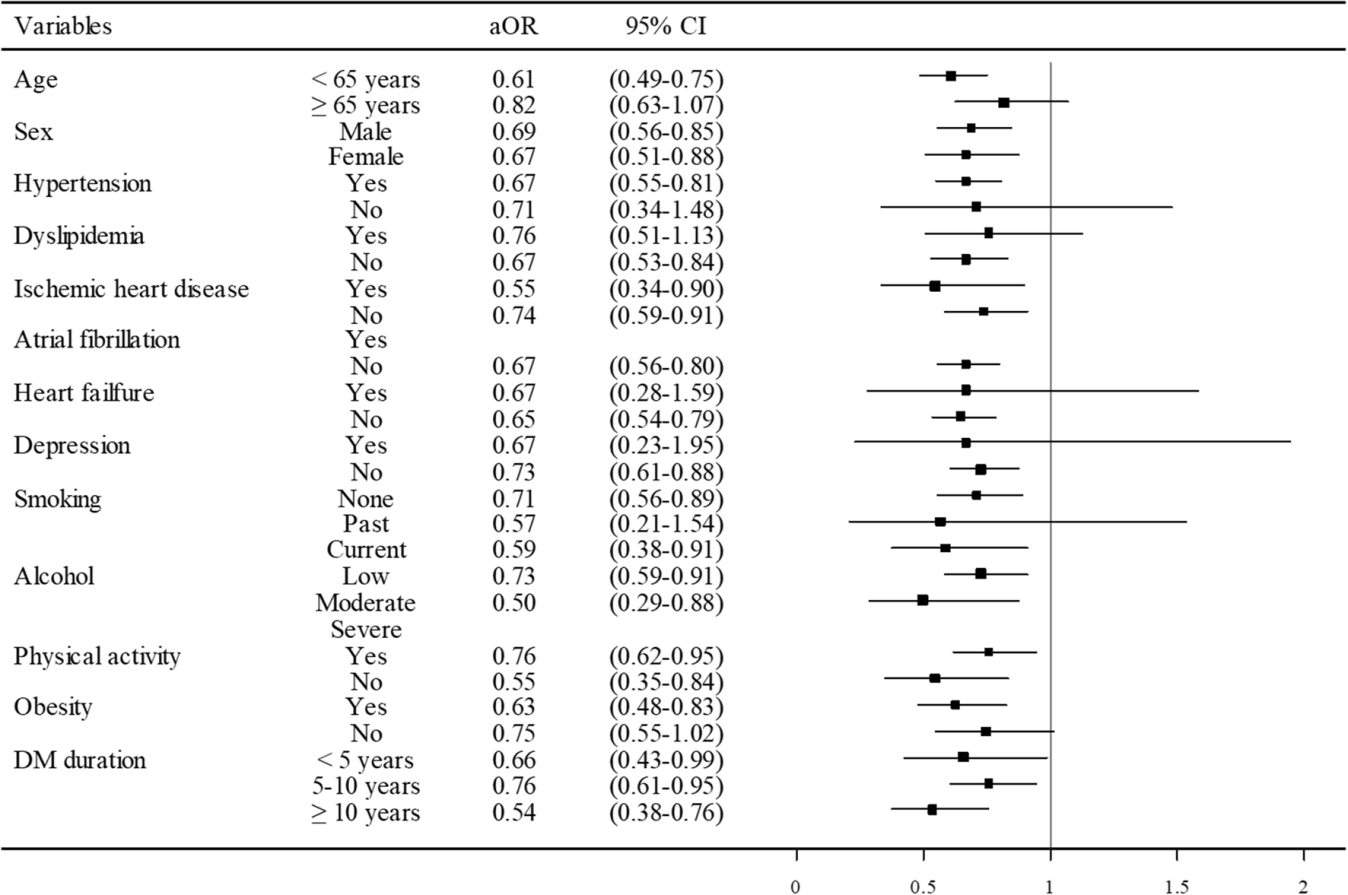
Odds ratios for ischemic stroke in the different subgroups of pioglitazone users. Boxes indicate the adjusted odds ratio (OR), and limit lines indicate the 95% confidence interval (CI). DM, diabetes mellitus

The effect of pioglitazone use on the risk of primary stroke was evaluated in a subsequent stratified analysis (Fig. 2). Pioglitazone use remained to be associated with a lower risk of stroke regardless of age, sex, DM duration, other medical comorbidities, and lifestyle risk factors, such as cigarette smoking, alcohol consumption, and physical activity. Of note, pioglitazone was shown to have a greater protective effect in patients with a higher risk of stroke, such as those with hypertension, IHD, longer duration of DM, obesity, current smoking, alcohol consumption, and no physical activity. These findings suggest that the beneficial effect of pioglitazone is more prominent for patients with DM with a higher risk of stroke. The subgroup analyses, which were conducted according to use of different medications, did not disclose any significant alteration in the observed effect of pioglitazone except on antiplatelets (Additional file 1: Table S1, Figure S1).

### Sensitivity analyses

A total of 4006 patients with ischemic stroke and 15,398 without ischemic stroke were matched in a 1:4 ratio. The demographic characteristics of the two cohorts were similar (Additional file 1: Table S2). After matching, the pioglitazone users still exhibited a significantly lower risk of primary ischemic stroke than did non-users (AOR 0.70, 95% CI, 0.58–0.84, Additional file 1: Table S3).

## Discussion

In this national, longitudinal nested case–control study, we evaluated 4,796 ischemic stroke cases and 23,980 control cases with matching variables including age, sex, time of DM diagnosis, and DM duration at a 1:5 ratio from the NHIS-DM cohort. We found that pioglitazone use was significantly associated with a reduced risk of primary ischemic stroke in patients with newly diagnosed DM. Of note, the protective effect of pioglitazone on incident ischemic stroke was greater in patients with known stroke risk factors, including hypertension, obesity, smoking, and no physical activity. These findings suggest that the increased risk of ischemic stroke in patients with DM might be weakened by pioglitazone use. To the best of our knowledge, this is the first large-scale population study to assess the efficacy of pioglitazone for primary stroke prevention in Asian patients newly diagnosed with DM.

### Previous studies on the cardiovascular effects of pioglitazone

Identifying the patients most likely to benefit from preventive care is a form of personalized medicine that is receiving attention in patient care. Previous studies found a protective effect of pioglitazone against recurrent stroke in patients with DM and prior stroke/ischemic attack. In the PROactive study, the stroke risk was reduced by 47% [11], and the Insulin Resistance Intervention After Stroke trial found that pioglitazone reduced the risk of ischemic events by 24% [9]. However, the association between pioglitazone use and its protective effect in primary stroke prevention in patients with DM was not clearly understood. In the PROactive study, there was no preventive effect against the development of stroke (HR 1.06; 95% CI 0.73–1.52, *p* = 0.767) [11]. Another population-based study in Taiwan also reported no protective effects of pioglitazone on ischemic stroke prevention [18]. However, these results should be interpreted carefully since they have short pioglitazone exposure periods of less than 5 years; the mean follow-up period was 2.9 years for the PROactive study and 672 days for the Taiwanese study. According to our results, the protective effect of pioglitazone against primary stroke increased with cDDD, and there was a significantly reduced risk in people with a DM duration of more than 10 years. This suggests that the effect of pioglitazone against primary stroke may not have been clearly revealed in the previous randomized controlled trials due to their short follow-up periods.

Recently, the effect of pioglitazone on stroke has been expanded to prediabetes or patients without a history of stroke. In the prediabetic population, good adherence to pioglitazone had a HR of 0.64 for stroke compared to placebo [19]. In a population-based study of 7,574 pioglitazone users and 32,337 non-pioglitazone users from the National Health Insurance Research Database, those who used pioglitazone has a lower risk of ischemic stroke (HR 0.78; 95% CI: 0.62–0.95) [12]. However, the study population was limited to patients with DM who had at least one cardiovascular risk factor, i.e., hyperlipidemia or hypertension, which may have caused selection bias. In this study, we found that pioglitazone use has a favorable effect on decreasing the risk of primary ischemic stroke in patients with newly diagnosed DM. In addition, the preventive impact was particularly significant with a high cDDD of pioglitazone use, or in patients with comorbid stroke risk factors, i.e., hypertension, IHD, obesity, smoking, alcohol use, and no physical activity.

There are concerns about the adverse effects of pioglitazone, such as increased fluid retention, weight gain, and heart failure [20]. Since the evidence is still equivocal, this may have affected the decision of physicians to actively prescribe the drug. However, recent studies reported that those risks may be minimized by lowering the dosage [21] or through combination therapy with a sodium-glucose co-transporter-2 (SGLT2) inhibitor or glucagon-like peptide -1 (GLP1) receptor agonist [22–24]. Pioglitazone added to metformin as a secondary oral antidiabetic agent has been reported to reduce the cardiovascular risk in patients with DM compared with sulfonylurea added to metformin [25]. Considering that most patients with DM receive additional medication other than metformin, pioglitazone, which is now generically available, may present a more affordable cost-effective cardioprotective option than SGLT2 inhibitor or GLP1 receptor agonist [26]. Clinical studies have shown no reduction or a slight reduction in major cardiovascular or cerebrovascular events through intensive glycemic control [27]. Rather, treatment of preexisting traditional cerebrovascular risk factors has consistently been associated with major cerebrovascular benefits in patients with DM [28–32]. Therefore, our results may provide an armamentarium to physicians committed to cardiovascular prevention in patient care and a rationale for using pioglitazone in patients at high risk of stroke.

### Mechanism of cardiovascular protection by pioglitazone

The exact mechanism underlying the association between pioglitazone use and reduced cardiovascular risk is uncertain. Thiazolidinediones are insulin-sensitizing antidiabetics agents that act through PPAR-γ to cause a durable improvement in glycemic control in patients with type 2 DM [33, 34]. In addition to glycemic control, pioglitazone also has a favorable effect on the plasma lipid profile; it reduces the levels of triglyceride and free fatty acid and increases the levels of high-density lipoprotein cholesterol [35]. More importantly, since PPAR-γ receptors are expressed in endothelial cells, arterial smooth muscle cells, and monocytes/macrophages, pioglitazone exerts direct anti-atherogenic effects on the arterial wall and has a direct anti-inflammatory and antioxidant effect against ischemic injury while also promoting neuronal regeneration [36–39]. A recent discovery suggests that pioglitazone may improve memory impairment by increasing low-density lipoprotein related receptor protein expression in the hippocampus, indicating that the effect of pioglitazone may extend to neuroprotection [40].

### Limitations

As this study was a population-based nested case–control study using a claims database, there were several limitations. The patients included in this study may not have taken their prescribed medication or the dose prescribed, leading to exposure misclassification. Although adjustment was made for various potential confounding parameters including baseline laboratory data, other glucose-lowering agents, and lifestyle information in the analyses, data on the severity of index stroke and hemoglobin A1c levels, which are strong prognostic factors in stroke patients, were not collected due to the lack of detailed clinical information. However, we further evaluated the interaction between pioglitazone and known risk factors and provided evidence that pioglitazone may be beneficial to people at risk. This was an observational study, not a randomized trial, and, therefore, we should also consider the possibility of hidden bias between patients who received pioglitazone and those who did not. Despite adjustment for some differences in baseline characteristics, residual unidentified confounders may influence the relationship between pioglitazone use and stroke risk. In this study, only a small proportion of patients received pioglitazone, which could result in biased estimates. Although administrative databases are increasingly used for clinical research, such studies are potentially susceptible to measurement errors arising from coding inaccuracies. To mitigate this weakness, we applied the definition that was validated from previous studies using the Korean NHIS cohort [10]. Lastly, we were unable to evaluate the potential adverse effects of pioglitazone such as weight gain, fluid retention, and fracture. Although we reported that there is a reduced risk of stroke according to the cDDD of pioglitazone, further research is needed to find the optimal dose that minimizes drug-related adverse events in addition to potential benefits. In addition, a large-scale prospective study is necessary to determine the long-term drug safety profile of pioglitazone.

## Conclusion

We found that pioglitazone use dose-dependently reduces the risk of primary ischemic stroke in patients with DM, particularly in individuals with a high risk of stroke. Large, prospective clinical studies are warranted to confirm the efficacy of pioglitazone use on prevention of primary ischemic stroke in patients with DM, especially in those who have additional risk factors of ischemic stroke.

## Supplementary Information

**Additional file 1**. Additional figure and tables.

## Data Availability

The data that support the findings of this study are NHIS claims data and are stored on a separate server managed by the NHIS. The datasets generated and analyzed during the current study are not publicly available due to restrictions by NHIS. Access to the data is regulated by Korean law and the Korean National Institute for Health and Welfare. Interested parties may submit a separate application to the NHIS for access. The NHIS accepts applications via their website (https://nhiss.nhis.or.kr) and require ethics approval from the researcher’s institutional review board and a study proposal.

## Abbreviations

aDCSI: Adapted Diabetes Complications Severity Index
AOR: Adjusted odds ratio
BMI: Body mass index
CCI: Charlson Comorbidity Index
cDDD: Cumulative defined daily dose
CI: Confidence interval
DM: Diabetes mellitus
GLP1: Glucagon-like peptide-1
IHD: Ischemic heart disease
ICD-10: International Statistical Classification of Disease and Related Health Problems: Tenth Revision
NHSP: National Health Screening Program
OR: Crude odds ratio
PPAR-γ: Proliferation activated receptor γ
PROactive: Prospective Pioglitazone Clinical Trial in Macrovascular Events
PSM: Propensity score-matching
SGLT2: Sodium-glucose co-transporter-2
SMD: Standardized mean difference
