# Supplementary material for: Pioglitazone use associated with reduced risk of the first attack of ischemic stroke in patients with newly onset type 2 diabetes: a nationwide nested case–control study
Source: Cardiovasc Diabetol. 2021 Jul 27;20:152. doi: 10.1186/s12933-021-01339-x (PMC8314540; doi:10.1186/s12933-021-01339-x)
Supplement: Supplementary file 1 — Additional file 1. Additional figure and tables. [file 12933_2021_1339_MOESM1_ESM.pdf]

Additional file 1: Table S1. ORs for primary ischemic stroke according to medications

| Medications | | | Case | Control | aOR (95% CI) | p-value |
|---|---|---|---|---|---|---|
| Metformin | Yes | Non-pio | 3,542 (93.8) | 13,433 (91.7) | 0.70 (0.58-0.84) | <0.001 |
| | | Pio | 234 (6.2) | 1,219 (8.3) | | |
| | No | Non-pio | 801 (99.1) | 1,943 (98.7) | 0.41 (0.14-1.21) | 0.106 |
| | | Pio | 7 (0.9) | 26 (1.3) | | |
| DPP-IV inhibitors | Yes | Non-pio | 893 (91.0) | 1,629 (87.0) | 0.75 (0.53-1.08) | 0.120 |
| | | Pio | 88 (9.0) | 243 (13.0) | | |
| | No | Non-pio | 3,428 (96.0) | 14,389 (95.7) | 0.75 (0.53-0.87) | 0.015 |
| | | Pio | 143 (4.0) | 642 (4.3) | | |
| Insulin | Yes | Non-pio | 1,251 (93.1) | 2,079 (90.1) | 0.64 (0.45-0.92) | 0.0146 |
| | | Pio | 93 (6.9) | 229 (9.9) | | |
| | No | Non-pio | 2,686 (95.6) | 10,607 (94.7) | 0.68 (0.53-0.87) | 0.0025 |
| | | Pio | 123 (4.4) | 589 (5.3) | | |
| SGLT-2 inhibitors | Yes | Non-pio | 8 (88.9) | 7 (77.8) | | |
| | | Pio | 1 (11.1) | 2 (22.2) | | |
| | No | Non-pio | 4,508 (94.9) | 22,119 (93.8) | 0.68 (0.58-0.81) | <0.001 |
| | | Pio | 241 (5.1) | 1,461 (6.2) | | |
| Sulfonylurea | Yes | Non-pio | 3,281 (93.5) | 11,187 (91.6) | 0.71 (0.59-0.86) | <0.001 |
| | | Pio | 228 (6.5) | 1028 (8.4) | | |
| | No | Non-pio | 1,096 (98.7) | 2,416 (97.7) | 0.47 (0.22-1.01) | 0.0521 |
| | | Pio | 15 (1.4) | 57 (2.3) | | |
| Alpha-glucosidase inhibitors | Yes | Non-pio | 130 (92.2) | 158 (91.3) | 0.32 (0.01-10.28) | 0.8261 |
| | | Pio | 11 (7.8) | 15 (8.7) | | |
| | No | Non-pio | 4,165 (95.0) | 19,155 (94.0) | 0.67 (0.56-0.81) | <0.001 |
| | | Pio | 220 (5.0) | 1,227 (6.0) | | |
| Statin | Yes | Non-pio | 2,353 (93.7) | 7,067 (91.2) | 0.70 (0.56-0.88) | 0.0025 |
| | | Pio | 158 (6.3) | 681 (8.8) | | |
| | No | Non-pio | 1,984 (96.3) | 5,165 (96.8) | 0.83 (0.55-1.23) | 0.3457 |
| | | Pio | 77 (3.7) | 173 (3.2) | | |
| Aspirin | Yes | Non-pio | 2,529 (94.4) | 7,215 (92.5) | 0.68 (0.53-0.87) | 0.0021 |
| | | Pio | 149 (5.6) | 585 (7.5) | | |
| | No | Non-pio | 1,803 (95.5) | 4,861 (95.3) | 0.79 (0.55-1.13) | 0.1958 |
| | | Pio | 86 (4.6) | 238 (4.7) | | |
| Antiplatelet | Yes | Non-pio | 473 (93.9) | 646 (91.2) | 0.48 (0.24-0.95) | 0.0354 |
| | | Pio | 31 (6.2) | 62 (8.8) | | |
| | No | Non-pio | 3,649 (95.2) | 15,925 (94.3) | 0.73 (0.59-0.89) | 0.0018 |
| | | Pio | 186 (4.9) | 967 (5.7) | | |

| | | | | | | |
|---|---|---|---|---|---|---|
| Anticoagulant | Yes | Non-pio | 21 (100.0) | 21 (91.3) | | |
| | | Pio | 0 (0.0) | 2 (8.7) | | |
| | No | Non-pio | 4,358 (94.9) | 21,115 (93.7) | 0.65 (0.55-0.78) | <0.001 |
| | | Pio | 234 (5.1) | 1,430 (6.3) | | |
| Antihypertensive agents | Yes | Non-pio | 3,318 (94.6) | 11,448 (93.0) | 0.68 (0.56-0.84) | <0.001 |
| | | Pio | 189 (5.4) | 867 (7.0) | | |
| | No | Non-pio | 1,012 (95.4) | 2,281 (95.4) | 0.66 (0.39-1.13) | 0.1269 |
| | | Pio | 49 (4.6) | 109 (4.6) | | |
| Antiarrhythmic agents | Yes | Non-pio | 258 (94.9) | 355 (93.7) | 1.25 (0.27-5.73) | 0.7762 |
| | | Pio | 14 (5.2) | 24 (6.3) | | |
| | No | Non-pio | 4,031 (95.0) | 18,201 (93.7) | 0.64 (0.53-0.77) | <0.001 |
| | | Pio | 214 (5.0) | 1,215 (6.3) | | |

ORs: odds ratios; CI: confidence interval; Non-pio: pioglitazone non-user; Pio: pioglitazone user; DPP-IV: dipeptidyl peptidase IV; SGLT-2: sodium-glucose co-transporter 2.

Additional file 1: Table S2. Patient characteristics in the PS-matched cohort

| | Before PS-matching | | | After PS-matching | | |
|---|---|---|---|---|---|---|
| | Ischemic stroke | | | | | |
| | No | Yes | SMD | No | Yes | SMD |
| Total n | 167,050 | 5,025 | | 15,398 | 4,006 | |
| Pioglitazone user | 10,727 (6.4) | 249 (5.0) | 0.063 | 1,053 (6.8) | 196 (4.9) | 0.083 |
| Age (mean (SD)) | 60.0 (8.0) | 62.6 (9.5) | 0.290 | 61.1 (8.7) | 61.8 (9.1) | 0.071 |
| Diabetes duration (mean (SD)) | 2,244.0 (1228.0) | 2,201.5 (1217.8) | 0.035 | 2,223.8 (1230.6) | 2,216.4 (1225.9) | 0.005 |
| Diabetes duration | | | 0.035 | | | 0.007 |
| <5 | 66,539 (39.8) | 2,051 (40.8) | | 6,193 (40.2) | 1,624 (40.5) | |
| 5-10 years | 75,135 (45.0) | 2,270 (45.2) | | 6,941 (45.1) | 1,801 (45.0) | |
| ≥10 years | 25,376 (15.2) | 704 (14.0) | | 2,264 (14.7) | 581 (14.5) | |
| Hypertension | 123,117 (73.7) | 4,113 (81.9) | 0.197 | 12,226 (79.4) | 3,218 (80.3) | 0.023 |
| Atrial fibrillation | 4,538 (2.7) | 367 (7.3) | 0.211 | 30 (0.2) | 30 (0.7) | 0.081 |
| Heart Failure | 16,312 (9.8) | 786 (15.6) | 0.177 | 1,553 (10.1) | 497 (12.4) | 0.074 |
| Ischemic heart disease | 38,165 (22.8) | 1,340 (26.7) | 0.089 | 3,179 (20.6) | 908 (22.7) | 0.049 |
| Dyslipidemia | 96,143 (57.6) | 2,546 (50.7) | 0.139 | 7,949 (51.6) | 2,038 (50.9) | 0.015 |
| CCI | | | 0.062 | | | 0.023 |
| 0 | 48,275 (28.9) | 1,500 (29.9) | | 4,754 (30.9) | 1,196 (29.9) | |
| 1 | 37,676 (22.6) | 1,233 (24.5) | | 3,568 (23.2) | 935 (23.3) | |
| 2 | 810,99 (48.5) | 2,292 (45.6) | | 7,076 (46.0) | 1,875 (46.8) | |
| DCSI | | | 0.171 | | | 0.115 |
| 0 | 153,912 (92.1) | 4,369 (86.9) | | 14,242 (92.5) | 3,574 (89.2) | |
| 1 | 8,995 (5.4) | 469 (9.3) | | 784 (5.1) | 305 (7.6) | |
| 2 | 4,143 (2.5) | 187 (3.7) | | 372 (2.4) | 127 (3.2) | |
| Depression | 19,293 (11.5) | 701 (14.0) | 0.072 | 1,853 (12.0) | 538 (13.4) | 0.042 |
| Statin | 93,833 (56.2) | 2,699 (53.7) | 0.049 | 8,074 (52.4) | 2,102 (52.5) | 0.001 |
| Aspirin | 87,206 (52.2) | 2,924 (58.2) | 0.121 | 8,120 (52.7) | 2,202 (55.0) | 0.045 |
| Antiplatelet | 19,478 (11.7) | 1,017 (20.2) | 0.236 | 1,155 (7.5) | 544 (13.6) | 0.199 |
| Anticoagulant | 2,759 (1.7) | 215 (4.3) | 0.155 | 20 (0.1) | 19 (0.5) | 0.063 |
| Antihypertensive agents | 110,467 (66.1) | 3,722 (74.1) | 0.174 | 10,873 (70.6) | 2,883 (72) | 0.030 |
| Antiarrhythmic agents | 14,521 (8.7) | 577 (11.5) | 0.093 | 1,285 (8.3) | 396 (9.9) | 0.054 |
| Alcohol use | | | 0.084 | | | 0.041 |
| Low | 119,883 (71.8) | 3,684 (73.3) | | 11,253 (73.1) | 2,928 (73.1) | |
| Moderate | 37,385 (22.4) | 979 (19.5) | | 3,210 (20.8) | 798 (19.9) | |
| Heavy | 9,782 (5.9) | 362 (7.2) | | 935 (6.1) | 280 (7.0) | |
| Smoking | | | 0.100 | | | 0.042 |
| None | 106,129 (63.5) | 3,120 (62.1) | | 9,869 (64.1) | 2,495 (62.3) | |
| Past | 24,121 (14.4) | 612 (12.2) | | 1,835 (11.9) | 479 (12.0) | |
| Current | 36,800 (22.0) | 1,293 (25.7) | | 3,694 (24.0) | 1,032 (25.8) | |
| Physical activity | | | | | | 0.044 |
| Yes (≥1 time per week) | 121,577 (72.8) | 3,331 (66.3) | 0.141 | 10,683 (69.4) | 2,698 (67.3) | |
| BMI | | | 0.065 | | | 0.044 |
| < 18.5 $kg/m^2$ | 1,226 (0.7) | 63 (1.3) | | 133 (0.9) | 52 (1.3) | |
| 18.5-22.9 $kg/m^2$ | 33,405 (20.0) | 1,078 (21.5) | | 3,139 (20.4) | 831 (20.7) | |
| 23-25 $kg/m^2$ | 42,019 (25.2) | 1,240 (24.7) | | 3,803 (24.7) | 986 (24.6) | |
| ≥ 25 $kg/m^2$ | 90,400 (54.1) | 2,644 (52.6) | | 8,323 (54.1) | 2,137 (53.3) | |

| | | | | | | |
|---|---|---|---|---|---|---|
| Fasting blood glucose (mg/dL) † | 135.3 (48.3) | 138.4 (54.1) | 0.062 | 137.7 (50.3) | 138.0 (52.4) | 0.006 |
| BP (mmHg) † | | | | | | |
| Systolic | 132.0 (17.1) | 134.9 (17.9) | 0.163 | 133.7 (16.8) | 134.6 (17.3) | 0.054 |
| Diastolic | 81.0 (10.7) | 82.0 (11.4) | 0.088 | 81.7 (10.6) | 82.0 (11.2) | 0.030 |
| Total cholesterol (mg/dL) † | 204.2 (41.5) | 205.1 (42.7) | 0.023 | 205.8 (41.8) | 205.4 (42.4) | 0.008 |
| Creatinine (mg/dL) † | 1.03 (0.97) | 1.02 (0.84) | 0.009 | 1.00 (0.85) | 1.02 (0.90) | 0.024 |
| Alpha-glucosidase inhibitors | 12,022 (7.2) | 424 (8.4) | 0.046 | 1,168 (7.6) | 339 (8.5) | 0.032 |
| DPP- IV inhibitors | 32,346 (19.4) | 1,272 (25.3) | 0.143 | 3,399 (22.1) | 990 (24.7) | 0.062 |
| Insulin | 35,136 (21) | 2,072 (41.2) | 0.447 | 4,548 (29.5) | 1,460 (36.4) | 0.147 |
| SGLT-2 inhibitor | 1,243 (0.7) | 47 (0.9) | 0.021 | 141 (0.9) | 37 (0.9) | 0.001 |
| Sulfonylurea | 112,032 (67.1) | 3,696 (73.6) | 0.142 | 10,958 (71.2) | 2,929 (73.1) | 0.044 |
| Metformin | 121,912 (73) | 3,989 (79.4) | 0.151 | 11,910 (77.3) | 3,146 (78.5) | 0.029 |

PS: propensity score; CCI: Charlson Comorbidity Index; DCSI: Diabetes Complications Severity Index; DPP-IV: dipeptidyl peptidase IV; SGLT-2: sodium-glucose co-transporter 2; SMD: standardized mean difference.

†Mean and standard deviation (SD) of the continuous independent variables in this study

Additional file 1: Table 3. Relationship of pioglitazone use with primary ischemic stroke stratified by cDDD and duration of pioglitazone use in patients with type 2 diabetes using sIPTW

| | Ischemic stroke | | | |
|---|---|---|---|---|
| | Yes | No | aOR (95% Cl) | P-value |
| Pioglitazone | | | | |
| Non-user | 3,810 (95.1) | 14,345 (93.2) | | |
| User | 196 (4.9) | 1,053 (6.8) | 0.70 (0.58-0.84) | <0.001 |
| Cumulative DDD of pioglitazone | | | | |
| Q1 (< 165 cDDD) | 61 (1.5) | 234 (1.5) | 0.95 (0.68-1.33) | 0.780 |
| Q2 (165-299 cDDD) | 52 (1.3) | 276 (1.8) | 0.77 (0.54-1.09) | 0.144 |
| Q3 (300-574 cDDD) | 47 (1.2) | 264 (1.7) | 0.65 (0.45-0.93) | 0.019 |
| Q4 (≥575 cDDD) | 36 (0.9) | 279 (1.8) | 0.45 (0.30-0.69) | <0.001 |
| Days of pioglitazone | | | | |
| 0 year | 3,810 (95.1) | 14,345 (93.2) | | |
| < 1 year | 72 (1.8) | 297 (1.9) | 1.00 (0.72-1.39) | 0.994 |
| 1-2 years | 59 (1.5) | 309 (2.0) | 0.74 (0.52-1.05) | 0.088 |
| 2-3 years | 26 (0.7) | 164 (1.1) | 0.61 (0.42-0.89) | 0.001 |
| 3 years ≥ | 39 (1.0) | 283 (1.8) | 0.47 (0.31-0.71) | <0.001 |

AOR: adjusted odds ratio; cDDDs: cumulative defined daily doses; CI: confidence interval; Q: quartile; sIPTW: stabilized Inverse Probability of Treatment weighting.

* Analysis was adjusted for the following covariates: DCSI, antiplatelets, and insulin.

Additional file 1: Figure S1. Forest plot of the ORs for primary ischemic stroke according to medications

| Medications | | aOR | 95% CI |
|---|---|---|---|
| Metformin | Yes | 0.70 | (0.58-0.84) |
| | No | 0.41 | (0.14-1.21) |
| DPP-IV inhibitors | Yes | 0.75 | (0.53-1.08) |
| | No | 0.75 | (0.53-0.87) |
| Insulin | Yes | 0.64 | (0.45-0.92) |
| | No | 0.68 | (0.53-0.87) |
| SGLT-2 inhibitors | Yes | | |
| | No | 0.68 | (0.58-0.81) |
| Sulfonylurea | Yes | 0.71 | (0.59-0.86) |
| | No | 0.47 | (0.22-1.01) |
| Alpha-glucosidase inhibitors | Yes | 0.32 | (0.01-10.28) |
| | No | 0.67 | (0.56-0.81) |
| Statin | Yes | 0.70 | (0.56-0.89) |
| | No | 0.83 | (0.55-1.24) |
| Aspirin | Yes | 0.68 | (0.53-0.88) |
| | No | 0.79 | (0.55-1.13) |
| Antiplatelet | Yes | 0.48 | (0.24-0.95) |
| | No | 0.73 | (0.59-0.89) |
| Anticoagulant | Yes | | |
| | No | 0.65 | (0.55-0.78) |
| Antihypertensive agents | Yes | 0.68 | (0.56-0.84) |
| | No | 0.66 | (0.39-1.13) |
| Antiarrhythmic agents | Yes | 1.25 | (0.27-5.73) |
| | No | 0.64 | (0.53-0.77) |

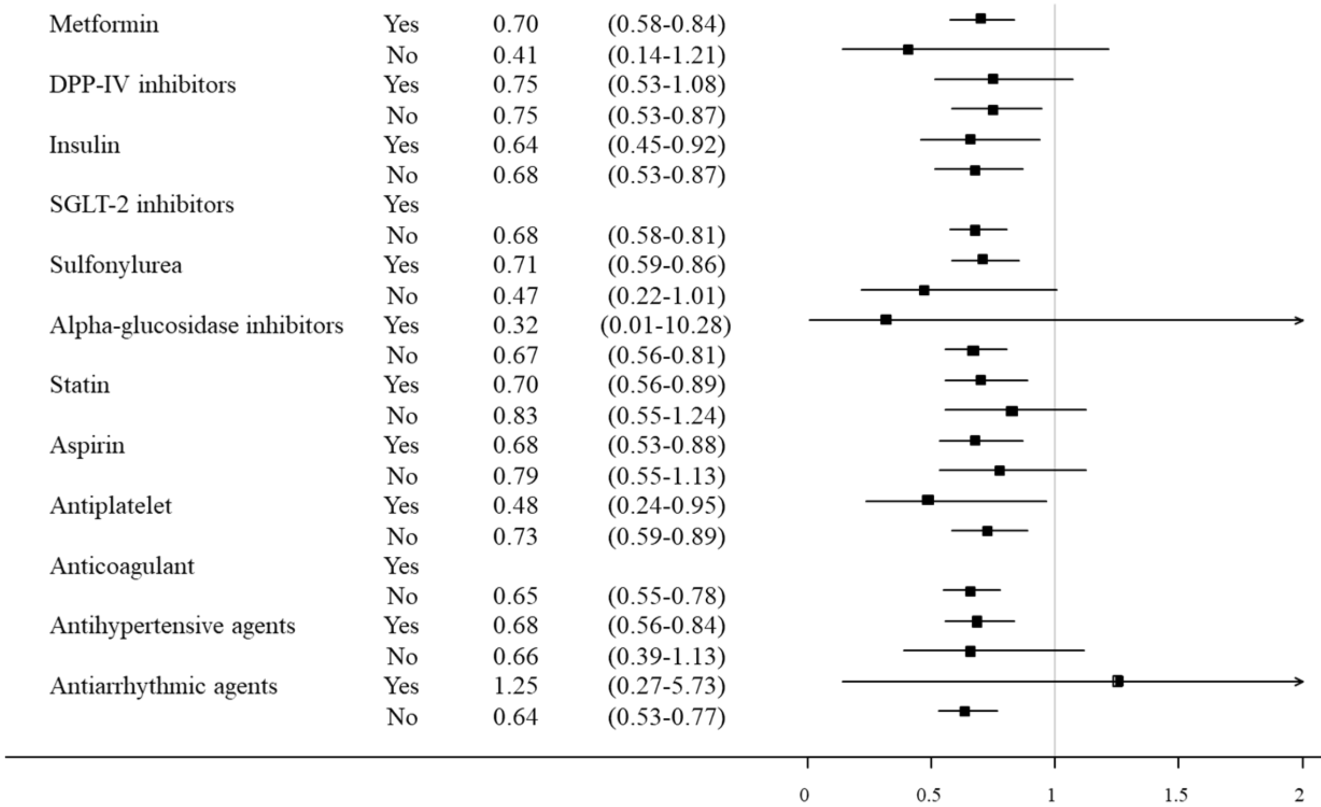


aOR: adjusted odds ratio; CI: confidence interval; DPP-IV: dipeptidyl peptidase IV; SGLT-2: sodium-glucose co-transporter 2.
